# Supplementary material for: No evidence that human GIGYF2 interacts with GRB10: implications for human disease
Source: Life Sci Alliance. 2025 Jun 16;8(9):e202503334. doi: 10.26508/lsa.202503334 (PMC12171015; doi:10.26508/lsa.202503334)
Supplement: Supplementary file 1 [file LSA-2025-03334_TableS1.docx]

**Table S1: Primers used in the study:**

| **Primer name** | **Species** | **Sequence (5´→ 3´)** |
| --- | --- | --- |
| Hs_mm_RT_*GRB10*_F | Human and mouse | GCGCCTTCAGGAGGAAGAC |
| Hs_mm_RT_*GRB10*_R | Human and mouse | CCAGCTGTTGTCATCCACACA |
| Hs_*SRSF2*_F1 | Human | TGGTCCTTTTTCCCCAAGTCC |
| Hs_*SRSF2*_R | Human | GTCCCGCACTCGTTCTCGAT |
| Hs_*SRSF6*_F1 | Human | ACGAGCTGAACGGCAAGGAG |
| Hs_*SRSF6*_R | Human | TCTTGCCAACTGCACCGACT |
